# On the Process-Related Rivet Microstructural Evolution, Material Flow and Mechanical Properties of Ti-6Al-4V/GFRP Friction-Riveted Joints

**DOI:** 10.3390/ma10020184

**Published:** 2017-02-15

**Authors:** Natascha Z. Borba, Conrado R. M. Afonso, Lucian Blaga, Jorge F. dos Santos, Leonardo B. Canto, Sergio T. Amancio-Filho

**Affiliations:** 1 Department of Materials Engineering, Federal University of São Carlos, São Carlos 310, Brazil; natascha.zocoller@hzg.de (N.Z.B.); conrado@ufscar.br (C.R.M.A.); leonardo@ufscar.br (L.B.C.); 2 Helmholtz-Zentrum Geesthacht, Center for Materials and Coastal Research, Institute of Materials Research, Materials Mechanics, Solid State Joining Processes, Geesthacht 21502, Germany; lucian.blaga@hzg.de (L.B.); jorge.dos.santos@hzg.de (J.F.d.S.); 3 Institute of Polymer Composites, Hamburg University of Technology, Hamburg 21073, Germany

**Keywords:** friction riveting, microstructural formation, microtexture, titanium alloy

## Abstract

In the current work, process-related thermo-mechanical changes in the rivet microstructure, joint local and global mechanical properties, and their correlation with the rivet plastic deformation regime were investigated for Ti-6Al-4V (rivet) and glass-fiber-reinforced polyester (GF-P) friction-riveted joints of a single polymeric base plate. Joints displaying similar quasi-static mechanical performance to conventional bolted joints were selected for detailed characterization. The mechanical performance was assessed on lap shear specimens, whereby the friction-riveted joints were connected with AA2198 gussets. Two levels of energy input were used, resulting in process temperatures varying from 460 ± 130 °C to 758 ± 56 °C and fast cooling rates (178 ± 15 °C/s, 59 ± 15 °C/s). A complex final microstructure was identified in the rivet. Whereas equiaxial α-grains with β-phase precipitated in their grain boundaries were identified in the rivet heat-affected zone, refined α′ martensite, Widmanstätten structures and β-fleck domains were present in the plastically deformed rivet volume. The transition from equiaxed to acicular structures resulted in an increase of up to 24% in microhardness in comparison to the base material. A study on the rivet material flow through microtexture of the α-Ti phase and β-fleck orientation revealed a strong effect of shear stress and forging which induced simple shear deformation. By combining advanced microstructural analysis techniques with local mechanical testing and temperature measurement, the nature of the complex rivet plastic deformational regime could be determined.

## 1. Introduction

The understanding of microstructural evolution is a key issue in welding and joining processes for similar and dissimilar joints with titanium alloys. This fundamental knowledge allows, for process optimization, a control of joint or weld mechanical properties. The influence of the joining process parameters on the final microstructure and its evolution in similar and dissimilar titanium alloy joints or welds have been widely explored in the literature [1,2,3,4,5]. Conventional welding processes of titanium alloys such as laser beam welding (LBW) [1,6], gas tungsten arc welding (GTAW) [4], high vacuum electron beam welding (EBW) [7], and solid-state welding processes such as ultrasonic welding [8] and friction stir welding (FSW) [2,4] have industrial applicability and thus have been deeply investigated. Welding of Ti-6Al-4V alloy is particularly challenging because this alloy is a bi-phase material at room temperature (α-Ti and β-Ti phases). Ti-6Al-4V welds generally present a complex final microstructure with combined equiaxed and acicular morphologies [9]. Consequently, a considerable number of studies on the topic have been published [1,4,5,8].

In GTAW of Ti-6Al-4V alloy, an increase in the arc current led to high heat input and consequently low cooling rates, favoring alpha grain coarsening, formation of a Widmanstätten microstructure, and brittle intermetallic compounds [3,4]. Danielson et al. [5] reported that the significant oxygen contamination on the surface of Ti-6Al-4V alloy GTAW welds led to a predominance of acicular morphology. For EBW, LBW and FSW, the refinement of the disoriented alpha acicular grains and the martensitic transformation originated, in the last case, from rotational speeds of up to 1000 rpm, welding speed of 400 mm/min (i.e., low energy input), and a fast cooling rate of 100 °C/s, improved the tensile strength of the welds and the hardness of the process-related microstructural zones [3,4,7]. Zhu et al. [8] demonstrated that by optimizing the welding pressure and time for ultrasonic welding of Ti-6Al-4V alloy and aluminum A6061, the hardness of both matrices increased with apparent diffusion occurring across the welding interface.

The microstructural evolution and final texture of Ti-6Al-4V alloy friction stir welds were recently investigated [10,11,12,13]. According to Fonda and Knipling [11], the predominant deformation during FSW is expected to be simple shear, which can be assessed by the planes/directions of the body centered cubic (bcc) structure attributed to the β-Ti phase. Zhang et al. [12] demonstrated that although the retained β-Ti phase can provide insights into the material flow, the fraction of this phase is usually too low in the final microstructure of Ti-6Al-4V alloy friction stir welds, hindering textural measurements. Thus, Mironov et al. [13] proposed the use of textural information from the major α-Ti phase to assess the simple-shear texture of the β-Ti phase by considering its [0001] and (11$\bar{2}$0) pole figures. This method has been successfully used for texture analysis in the near-α titanium friction-stir welds as well [14].

Nevertheless, few studies have addressed the effect of microstructural transformations on the local and global mechanical performance of hybrid joints of Ti-6Al-4V alloy and polymers or polymeric composites. Recently, Kashaev et al. [15] compared laser riveting to conventional riveting combined with adhesive bonding and laser riveting with surface structuring to join a carbon-fiber reinforced polymer and Ti-6Al-4V alloy. Although laser riveting led to high stiffness under quasi-static loading, among the compared joints, higher fatigue life was achieved using adhesive bonding and surface structuring. The lower fatigue performance of the laser-riveted specimen was correlated with the process-generated morphology gradient in the weld microstructure; a stress concentration effect associated with changes in the local strength from globular to acicular microstructure was responsible for decreasing the dynamic mechanical performance of the joints.

**Figure 1:**
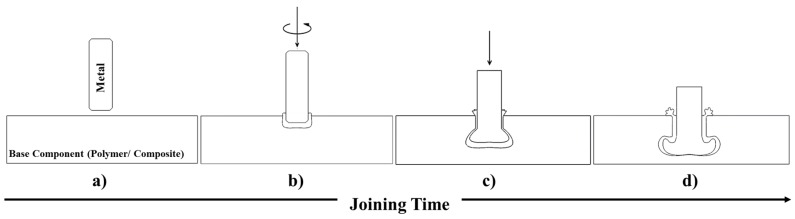
Steps of the FricRiveting process in metallic-insert joints. (**a**) Positioning of the joining parts; (**b**) insertion of the rotating metallic rivet into the polymeric plates (frictional phase); (**c**) plastic deformation of the rivet tip by increasing the axial force (forging phase); (**d**) joint consolidation.

Friction riveting (FricRiveting) is an alternative mechanical fastening technology for multi-material structures that uses frictional heat and pressure to plasticize and deform a cylindrical metallic rivet into a polymeric part, leading to strong mechanical interlocking. Concomitantly, processual heat softens the polymeric part (temperature above glass transition or melting point of the polymer), which will consolidate around the rivet, spawning adhesion forces at the interface [16]. A schematic description of the process in its basic configuration (i.e., metallic-insert joint) is shown in Figure 1. Different aspects of the FricRiveting were investigated and described in our previous publications. These range from a general description of the process, feasibility studies and microstructural features of the joints [16,17,18,19,20], process optimization for distinctive material combinations [19,20], the mechanical behavior under tensile [19,20] and shear loading [18,21], as well as an introductory study on the microstructural evolution of commercially pure titanium alloy grade 3/short-carbon-fiber reinforced polyether ether ketone (CF-PEEK) friction-riveted joints [17]. In the later publication, the microstructural evolution in the deformed rivet tip was investigated by electron backscatter diffraction (EBSD) to analyze process-related metallurgical phenomena through the rivet length. However, the authors did not address the correlation between the rivet microstructure, plastic deformation regime of the rivet and joint local mechanical performance.

We have demonstrated the feasibility of FricRiveting in Ti-6Al-4V alloy and glass-fiber reinforced thermoset polyester (GF-P) [18], material currently used in structural profiles of civil infrastructures such as bridges [22]. The correlations between joining parameters, energy input, process temperature and rivet deformation were determined. However, neither metallurgical transformations in the Ti-6Al-4V joining partner nor their correlations with rivet plastic deformation, metallic flow or joint mechanical performance were addressed.

Therefore, the present study aims to understand the process-related microstructural transformation of Ti-6Al-4V alloy in hybrid friction-riveted joints with pultruded glass-fiber-reinforced thermoset polyester (GF-P) and its correlation with the rivet plastic deformation regime and joint quasi-static mechanical behavior. The metallic-insert friction-riveted joint has been mechanically evaluated by connecting with an AA2198 gusset with bolt nuts and washers. This approach was adopted in order to simulate the real connections in future structural applications such as emergency bridges, in which metallic connectors may be applied to assemble pre-friction-riveted joints of bridge structural profiles made of single composite parts [21]. Thus, the assembly of pre-joined profiles can be performed readily on site and enables also the reassembly of the structure for maintenance purpose. Nonetheless, the gusset is responsible only for the load transfer and has no influence on the actual friction-riveted joint. In our previous publications [19,20], we have shown that joint strength is strongly dependent of the mechanical anchoring attributed to the rivet tip widening, and of the polymeric part to a lesser extent. Although complex, microstructural transformation in the GF-P part does not play an important role in dictating quasi-static failure of friction-riveted joints, as shown in a previous publication [18]. Therefore, the transformations in the GF-P microstructure will be discussed in a separate manuscript. In the present work, strong friction-riveted joints, displaying similar mechanical performance as state-of-the-art bolted joints, were selected for detailed microstructural and rivet plastic deformation analyses. Selected representative results on process temperature and the local mechanical properties (Vickers microhardness) are presented to support microstructural observations. High shear rates, process temperatures and cooling rates during FricRiveting induced a complex microstructural change in the Ti-6Al-4V rivet comprising Widmanstätten and martensitic structures formed in the plastically deformed rivet tip. These results were further investigated by assessing rivet texture to support the understanding of the rivet plastic deformation regime during the joining process and to help elucidate the joint mechanical performance.

## 2. Materials and Methods

### 2.1. Materials

**Figure 2:**
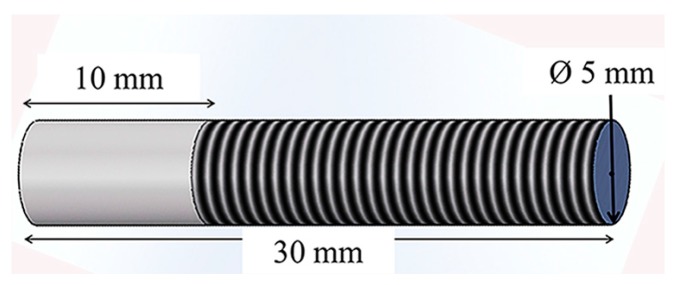
Dimensions of the threaded rivet (M5) used in this work.

Extruded rods of Ti-6Al-4V alloy were used for manufacturing cylindrical rivets 5 mm in diameter and 30 mm long. Figure 2 schematizes the rivet profile, which consists of a 10-mm-long unthreaded tip and full-threaded (DIN-M5) length of 20 mm. The threaded portion of the rivet was designed to be used during the assembly and clamping of overlap joints with nuts and washers.

**Figure 3:**
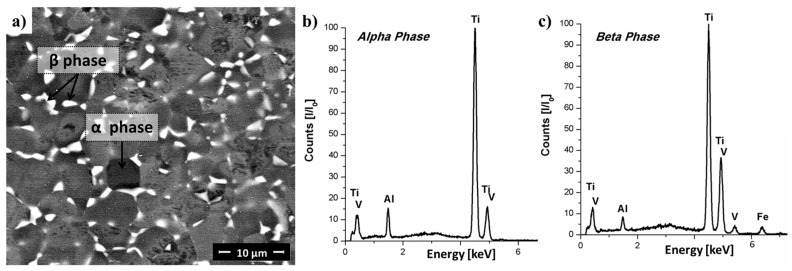
(**a**) Microstructure of Ti-6Al-4V alloy rivet material showing α equiaxed grains with β-phase in the α grain boundaries; energy dispersive spectroscopy (EDS) spectra of semi-quantitative composition of the (**b**) α-Ti and (**c**) β-Ti phases.

The microstructure of Ti-6Al-4V base material comprises two phases with an equiaxed β-Ti (bcc) phase (grain size of 0.6 ± 0.2 µm) distributed in the grain boundaries of α-Ti (hexagonal close packed, hcp) phase (grain size of 4.5 ± 0.9 µm), as displayed in Figure 3a. Figure 3b,c show energy dispersive spectroscopy (EDS) results. Table 1 lists the experimental chemical composition of the Ti-6Al-4V alloy used in this work. The main properties of this alloy are summarized in Table 2.

The composite part was a 10 mm thick pultruded glass-fiber-reinforced thermoset polyester with 50 wt. % nominal E-glass fiber content, with a stacking sequence of (0°,90°)/(±45°)/(0°,90°), supplied by Fiberline Composites A/S, Denmark. Relevant thermal and mechanical properties of GF-P are listed in Table 2.

### 2.2. Joining Procedure

Joints were produced by time-controlled mode in a friction welding machine (RSM 400, Harms & Wende, Hamburg, Germany). Two joint configurations were produced. While for microstructural analysis and for the investigation of local properties, metallic-insert joints of square (36 mm × 36 mm) GF-P plate were used, for lap shear testing an external metallic plate was used as a gusset to transfer the load during the test. After an exploratory study and joint optimization [18], low (LEI) and a high (HEI) energy input conditions were selected to evaluate the effect of heat generation on the final metallic microstructure, plastic deformation of the rivet tip, and joint mechanical performance. Table 3 lists the joining parameters used in this work.

The energy input for the LEI and HEI joining conditions was estimated using the mechanical work, to evaluate the effect of heat generation on the plastic deformation of the rivet tip using Equation (1) [25]. The total energy input (*E*_total_) comprises the frictional contribution (*E*_fr_), which is the product of angular velocity (ω) and frictional torque (*M*_z_), and a normal deformation contribution (*E*_ax_), which is a product of the axial force (*F*) and the burn-off rate (*υ*_0_) (the amount of rivet insertion and deformation, obtained from the rivet displacement monitoring curve). The experimental input data needed for the calculation were acquired from the monitoring system of the RSM 400 equipment and a torque sensor (model 9049, Kistler, Switzerland).

(1) $$E_{\mathrm{total}}=E_{\mathrm{fr}}+E_{\mathrm{ax}}=\int \text{​}M_{\text{z}}\times \omega \;dt+\int \text{​}F\times \upsilon_{0}\;dt\;\left[J\right]$$

**Figure 4:**
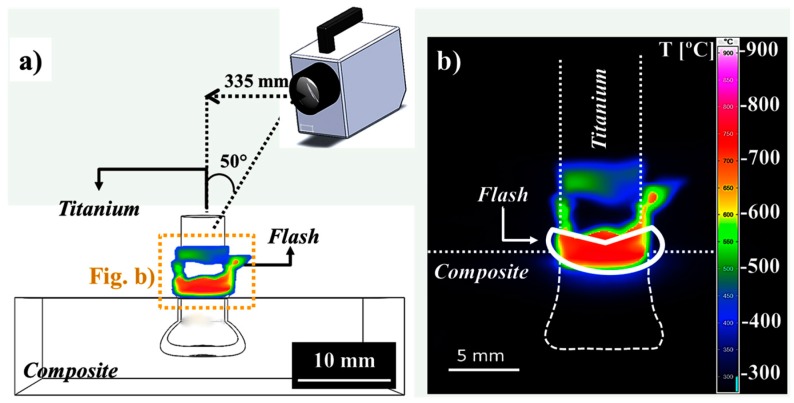
(**a**) Schematic configuration of infrared thermography showing the distance between the thermocamera and the joint and the flash where the temperature was recorded; (**b**) example of a thermogram showing the maximum process temperature. The continuous white line in (**b**) delimits the monitored area.

Infrared thermography (infrared camera ImageIR^®^8800, InfraTec GmbH, Dresden, Germany) was used to monitor the process temperature during the joining within the temperature calibration range of 300 to 1300 °C at a data acquisition rate of 20 Hz. Measurements were carried out on the flash material expelled from the composite plate during rivet insertion. Figure 4a illustrates the measurement configuration and Figure 4b shows a thermogram in which the measured area is indicated by a continuous white line. The peak temperature in the selected measured area was reported as the process temperature. The cooling rate was calculated from the maximum temperature to the minimum value from the calibration range (300 °C) adopting a linear fitting approach commonly used in the literature [26]. This is a simplification which does not consider variations in cooling rate during the cooling regime of friction-riveted joints.

### 2.3. Experimental Procedure

**Figure 5:**
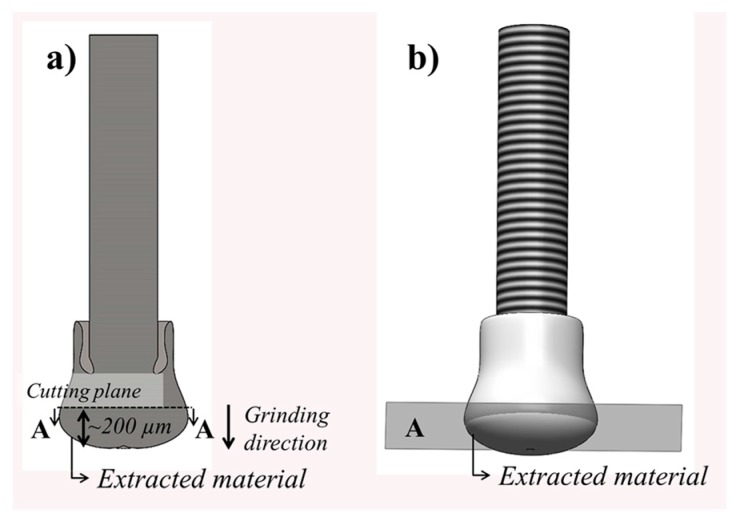
(**a**) Schematic cross-sectional view of the rivet detailing the grinding direction and the selected region of material extraction for TEM analysis (also shown in perspective in (**b**)).

The features of the deformed rivet tip (joint anchoring zone) were first assessed using a Leica DM IRM light optical microscope (LOM) (Leica Microsystems, Wetzlar, Germany). The joints were cut near the middle cross-section and prepared following the standard procedures for metallographic sample preparation. The details of the process-related microstructural changes in the metal were assessed by a scanning electron microscopy with secondary electrons (SEM, FEI Inspect S50, FEI, Hillsboro, OR, USA) and transmission electron microscopy (TEM, FEI TecnaiTM G2 F20, FEI, Hillsboro, OR, USA). For the SEM analysis, samples from the LOM analysis were chemically etched with Kroll reagent (100 mL distilled water, 2 mL hydrofluoric acid and 5 mL nitric acid), at room temperature for 15 s. The size and aspect ratio (length, l, as a function of width, w; w/l) of the grains were measured and calculated using Image J software (Image Pro Plus, FIJI, Bethesda, MD, USA) based on the ASTM E112 standard. For TEM analysis, material was carefully extracted from the deformed rivet tip by mechanical cutting under cooling, as schematically shown in Figure 5a,b. Thin foil samples were prepared by grinding down to a thickness of approximately 150 µm and applying twin-jet electro-polishing in a solution of 10 mL of sulfuric acid and 90 mL of methanol. The electro-polishing procedure was carried out using a Struers Tenupol-5 device operating at voltage of 15–20 V and temperature of −40 °C. Additionally, the local chemical composition of the metallic phases was assessed by energy dispersive spectroscopy (EDS, EDAX, Weiterstadt, Germany).

Microtexture analysis by electron backscattered diffraction (EBSD) was carried out in a FEI Inspect S50 microscope. The specimens were prepared following the same procedure adopted for LOM with an additional polishing step with colloidal silica slurry (0.02 µm). The samples were further electropolished in an electrolyte solution (940 mL acetic acid, 60 mL perchloric acid) using voltage of 35 V and current of 0.5 A for 5 min at 10 °C [27]. EBSD maps were acquired using a spatial step size of 0.2 µm and 0.1 µm, depending on the level of microstructural refinement. The average confidence index varied between 0.2 and 0.46, depending on the step size and the microstructure.

The qualitative identification of the phases in the Ti-6Al-4V deformed rivet tip was performed by X-ray diffraction (XRD) using a Siemens D5005 diffractometer with Cu Kα radiation at 40 kV and 40 mA at 2°min^−1^. The analysis was performed over the range of 30° < 2θ < 90°. XRD samples were extracted from the same region as the TEM specimens, as displayed in Figure 5a,b.

Vickers microhardness testing was employed to qualitatively identify the process-related metallurgical transformations and establish the limits of microstructural zones in the metal. Microhardness tests were performed in a Zwick/Roell-ZHV tester with an indentation load of 0.5 HV (4.9 N) and a holding time of 15 seconds; the distance between the indentations was set to 300 μm.

**Figure 6:**
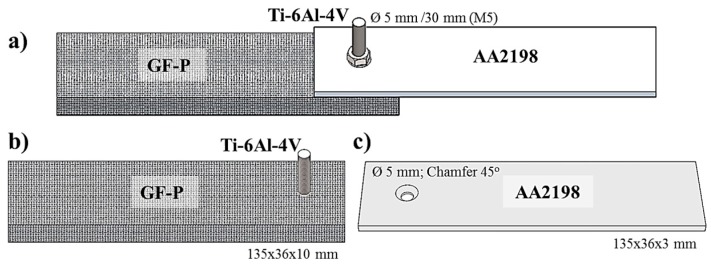
(**a**) Geometry (schematic representation) of a single overlap specimen for lap shear testing; (**b**) Ti-6Al-4V/GF-P friction-riveted joint and (**c**) gusset plate of AA2198.

The global mechanical performance of the joints was assessed by lap shear testing and carried out using a Zwick 1478 universal testing machine (Zwick/Roell, Ulm, Germany) equipped with a load cell of 100 kN and crosshead speed of 2 mm/min at room temperature (21 °C). Five replicates for each processing condition described in Table 3 were tested. Single overlap specimens were prepared based on ASTM D 5961 M-08, as schematically illustrated in Figure 6a. Ti-6Al-4V was pre-riveted in the GF-P plate (Figure 6b). Aluminum sheets (AA 2198 alloy, 3 mm thickness, see properties in Table 2) perforated with a through-hole diameter of 5 mm [18] were used as a gusset to transfer the load to the joint during the test (Figure 6c). This element is external to the friction-riveted joints. This procedure was adopted to simulate the real connections in a future structural application, in which metallic connectors may be applied to assemble butt joints of bridge composite profiles, as previously mentioned [21]. Finally, the two overlapping parts were tightened together using stainless steel nuts and washers. A clamping torque of 5 Nm was applied with a torque wrench to tighten the joint with M5 nuts and washers following the procedure described in [21]. Mechanical performance is reported as ultimate lap shear force (ULSF); stress values were not calculated because of the complexity in determining the bearing area between the deformed rivet and composite part.

## 3. Results and Discussion

### 3.1. Quasi-Static Global Mechanical Performance of the Joints

**Figure 7:**
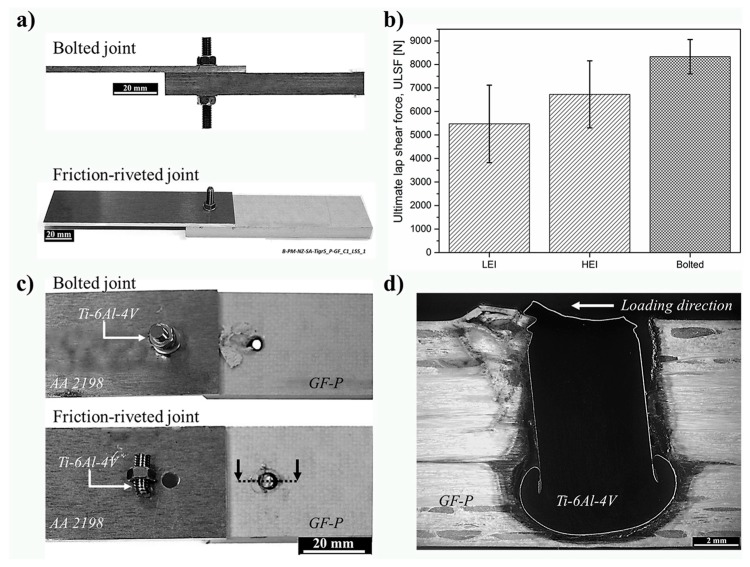
(**a**) Overview of an overlap Ti-6Al-4V/GF-P friction-riveted and bolted joints; (**b**) average ultimate lap-shear forces of friction-riveted joints produced under low (LEI) and high (HEI) energy inputs and state-of-the-art bolted joints; (**c**) example of fracture surfaces of a HEI friction-riveted and bolted joints; (**d**) cross-section of fractured friction-riveted joint from (**c**), showing the loading direction.

Lap shear testing was performed to evaluate the quasi-static global mechanical performance of friction-riveted LEI and HEI joints and for comparison purposes with state-of-the-art bolted joints. Figure 7a shows the friction-riveted and bolted joint configurations. Figure 7b compares the average ULSF of LEI and HEI friction-riveted joints with bolted joints.

The friction-riveted joints exhibited an ultimate lap-shear force (ULSF) of 5.5 ± 1.9 kN for LEI and 6.8 ± 1.7 kN for HEI conditions. Although the average ULSF of HEI specimens are slightly higher, the values of quasi-static lap shear performance are statistically similar for the range of joining parameters selected. When comparing friction-riveted specimens with state-of-the-art bolted joints with an average ULSF of 8.7 ± 0.5 kN, an average decrease of 22%–37% in ULSF for friction-riveted joints was observed. However, the strongest HEI friction-riveted specimens achieved an ULSF of up to 8.3 kN, which is within the standard deviation of bolted joint strength. Both joints failed initially through bearing of the composite and finally through shearing of the metallic rivet shaft, as shown in Figure 7c. Figure 7d shows a typical cross-section of the fractured friction-riveted joint where cracks, due to the composite bearing, can be identified in the upper left side of the joint and the remained failed rivet. Due to the notch effect of the rivet threads and the high stress concentration in the first two rivet fillets, the shear strength of the joint was lower than the nominal shear strength of Ti-6Al-4V (550 MPa, Table 2) leading to a final fracture through the rivet (Figure 7d). The fracture micro-mechanisms, which lead to the final failure observed, involve a complex combination of ductile and brittle failure of the metal and will be not addressed in this work.

Similar results were reported by Blaga et al. [21] for mechanical and failure behaviors of friction-riveted and bolted lap-shear joints of glass-fiber reinforced polyetherimide (PEI) joined with aluminum gussets and commercially pure titanium grade 2 rivets and bolts. After optimizing the lap shear strength of friction-riveted joints by design of experiments, the authors demonstrated that friction-riveted joints achieved strengths of up to 20% higher than bolted connections. Although the mechanical performance of current friction-riveted joints is statistically comparable to bolted joints, an improvement in ULSF may be achieved with further process optimization.

### 3.2. Temperature History

**Figure 8:**
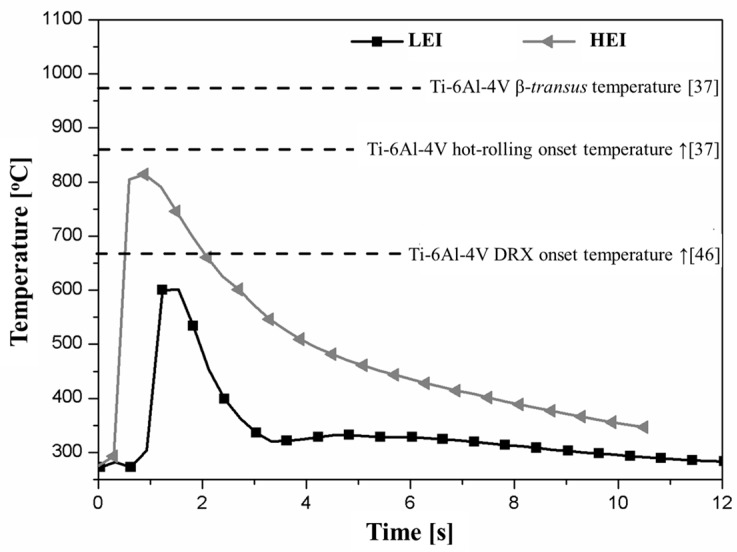
Process temperature evolution of low (LEI) and high (HEI) energy input joints measured by infrared thermography and the comparison with important onset transformation temperatures of the titanium alloy.

The temperature history is a key factor to understand the process-related microstructural transformations in the joining parts and consequently the plastic deformation regime in the rivet tip [28]. According to Amancio-Filho and dos Santos [29], several possible static and dynamic metallurgical phenomena, including hardening and annealing processes, can occur in the metallic rivet. Figure 8 presents an example of the evolution of the FricRiveting process temperature on the expelled polymeric flash material for friction-riveted joints produced with low and high energy inputs. The thermal data indicate an increase of approximately 65% in the peak temperature (from 460 ± 130 °C to 758 ± 56 °C) when the energy input was increased by changing the friction time from 1.0 to 1.2 s. Although the maximum temperatures were achieved during the friction phase for LEI and HEI conditions, joints of HEI expelled polymeric flash material earlier in the process, achieving the peak temperature at around 0.9 s; for joints of LEI, a long time was required to form the flash—around 1.2 s—and consequently to be detected by the infrared thermo-camera. The inhomogeneous heat dissipation in the pultruded composite may explain this effect.

The average results of temperature measurements are summarized in Table 4. The heat dissipation regime in the joint area is still not well understood in FricRiveting. Considering that forced cooling was not applied in the joining process, it is currently assumed that the heat dissipation probably relates mostly to the thermal properties of the joint materials and content or distribution of the fiber in this area along with the amount of heat generated during the frictional phase. It is known that Ti-6Al-4V and GF-P exhibit low thermal conductivity (Table 2). Moreover, the inhomogeneity of the fiber content and distribution throughout the GF-P thickness—owing to the pultrusion manufacturing process of GF-P—probably causes an inhomogeneous heat distribution through the joint, increasing the complexity of the analysis. Joints produced under the LEI condition (see example of a replicate in the square-bullet point curve in Figure 8) revealed a higher average cooling rate (178 ± 15 °C/s), whereas those produced under the HEI condition (see example of a replicate in the triangle bullet point curve in Figure 8) resulted in a lower cooling rate (59 ± 15 °C/s). Because HEI joints underwent a higher process temperature, the heat generation was higher and the expelled flash was previously exposed to the hot metallic surface for a longer period, requiring a longer time to dissipate the heat; this relationship may explain the lower cooling rates. Although in this work the cooling rates of HEI joints were considered slower than the LEI joints, these values are extremely fast compared to other friction-based processes such as friction spot joining [30] (cooling rates between 17 °C/s and 97 °C/s).

**Figure 9:**
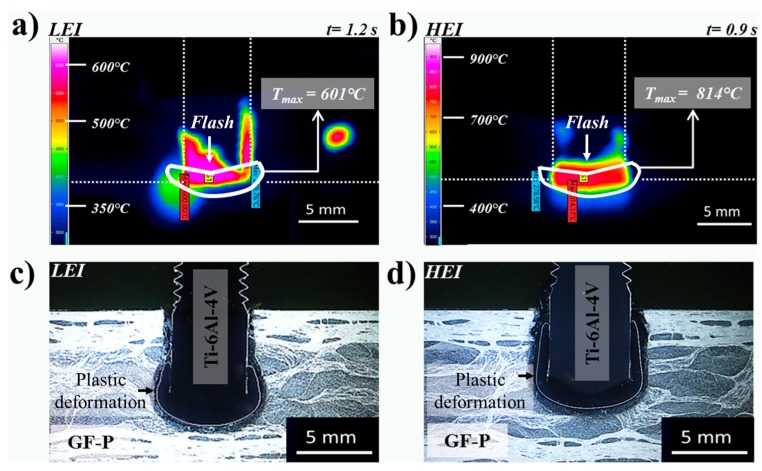
Infrared thermographs showing the peak process temperature (*T*_max_) and flash material for (**a**) LEI and (**b**) HEI conditions. Cross-sectional view of (**c**) LEI and (**d**) HEI Ti-6Al-4V/GF-P joints showing the plastic deformation of the rivet tip.

The average peak temperatures reached values of only up to 28%–45% of the Ti-6Al-4V alloy melting temperature (1665 °C) and were below its hot-rolling processing temperature (860–980 °C) [24]. Consequently, melting and plastic deformation of the metallic riveted should be absent. Whereas the former is true, extended plastic deformation of the rivet tip was observed for the studied specimens (see example in Figure 9c,d). This finding may indicate that the real temperature developed in the rivet tip underwent higher values than that measured by infrared thermometry. Figure 9a,b graphically show the maximum temperatures—i.e., peak process temperature—achieved for a typical joint for each joining condition, whereas Figure 9c,d emphasize the plastic deformation in the rivet tip as a function of process temperature. By increasing the energy input, higher process temperatures are achieved, leading to a higher volume of plasticized metal in the rivet tip (i.e., an increase in formability in the rivet tip).

The thermal data were also in the theoretical range of the dynamic recrystallization (DRX) of Ti-6Al-4V alloy (660–825 °C) and near the β-transus temperature (995 °C) [31]. As reported by Kitamura et al. [32] for FSW of Ti-6Al-4V—a friction-based joining process exhibiting similar severe deformation conditions as in FricRiveting—a non-equilibrium β-transus temperature was measured at values lower than 949 °C. Other authors reported temperature ranges even lower, in which the end of the β-to-α transformation was between 670 and 690 °C for cooling rates of 50–10 °C/min [33,34]. In FricRiveting, a similar decrease in the onset β-transus temperature may be expected. Considering the process-related large deformation and heat generation, the required energy to reach the necessary enthalpy to transform the α-Ti phase into the β-Ti phase is expected to be reduced, favoring a decrease of the onset β-transus temperature [32]. Evidence from microstructural analysis (see Section 3.3) helps to support this assumption.

**Figure 10:**
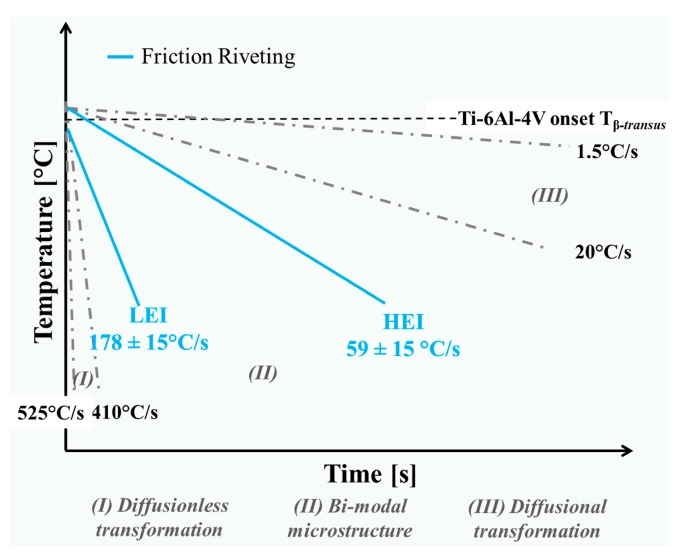
Schematic continuous cooling diagram for Ti-6Al-4V alloy including the calculated cooling rate of friction-riveted joints (adapted from [35]).

Furthermore, high process temperatures, plastic deformation and cooling rates may induce changes in phase morphology—e.g., the formation of acicular and equiaxed [24] grains—in the microstructure of Ti-6Al-4V rivets. Ahmed and Rack [35] reported that continuous cooling transformation (CCT) diagrams in α + β titanium alloys can provide valuable data to increase understanding of the formation of different phase morphologies in Ti-6Al-4V rivets. The authors have shown that to attain a range of microstructures, the material has to experience different values of cooling rates [32]. Cooling rates calculated for friction-riveted joints ranging between 59 ± 15 °C/s and 178 ± 15 °C/s (Table 4) are schematically compared to the previously documented CCT diagram in Figure 10.

As can be observed in the CCT diagram (Figure 10), three types of microstructure—fully martensitic or diffusionless structure, diffusional structure and a combination of these two (bi-modal)—can be formed [35]. The calculated cooling rates for the selected friction-riveted joints lie in a bi-modal microstructure field, which may indicate the presence of equiaxed grains combined with acicular structures. The acicular structures can be formed as a result of either diffusionless (metastable α′ martensite) or diffusional (Widmanstätten) transformations [36]. These phase morphologies give rise to different mechanical properties; equiaxed morphology leads to higher ductility, whereas lamellar structure leads to higher tensile strength [2,3]. The changes in local mechanical properties in the metallic rivet will be discussed in Section 3.4.

Based on the temperature assessment, a considerable number of phenomena can occur in the metallic component in the joint area of Ti-6Al-4V/GF-P friction-riveted joints. These may include dynamic recrystallization and phase transformations. The predominance of one morphology over the other can influence the plastic deformation regime of the rivet tip and therefore affect the local and global mechanical performance of the joints. Thus, the metallurgical transformations in FricRiveting must be deeply investigated. The following sections address the outcomes of the process-related microstructural changes on the local mechanical properties of the joints and discuss the primary phenomena observed in the titanium joining part.

### 3.3. Ti-6Al-4V Rivets: Microstructural Evolution in the Joining Area

Considering that both HEI and LEI specimens presented similar process-related microstructural changes in the rivet, a specimen with high mechanical performance from joining condition HEI (Section 3.1) was chosen for detailed microstructural characterization. Because of the high process temperature (758 ± 56 °C) and fast cooling rate (59 ± 15 °C/s), pronounced process-related microstructural changes are expected to occur during the joining process.

**Figure 11:**
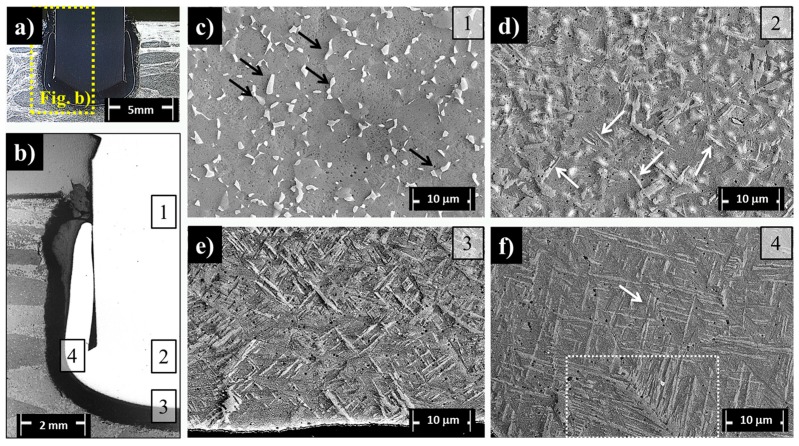
(**a**) Full cross-section view of a typical Ti-6Al-4V/GF-P joint; (**b**) detailed view of half section of the joint in (**a**), where the microstructural analysis was performed; regions (1–4) are detailed in (**c**–**f**) figures, respectively (HEI joining conditions: rotational speed = 9000 rpm, friction time = 1.2 s and forging time = 1.2 s). In (**f**), Widmanstätten and martensitic structures are depicted by a dash-line rectangle and an arrow, respectively.

The typical microstructures of relevant regions (Regions 1–4) (Figure 11a,b) over the cross-section of the Ti-6Al-4V/GF-P joint are detailed by SEM analysis in Figure 11c,f. The results of average grain size measurements are compiled in Table 5. Region 1, located far from the plastically deformed metallic rivet tip (the anchoring zone, Figure 11c), is fully composed of equiaxed primary alpha phase (α-Ti phase) with an average grain size of 5.4 ± 1.8 µm. An elongated beta phase (β-Ti phase, indicated by black arrows in Figure 11c) is precipitated in the α-Ti grain boundaries; they have a phase width of 0.9 ± 0.2 µm and an aspect ratio of 0.4 ± 0.1 (Table 5). This phase morphology is similar to that observed in the as-received metallic rivet (Figure 3). However, the larger average grain size in Region 1 (w_R1-α-grain_ = 5.4 ± 1.8 µm and w_R1-β-grain_ = 0.9 ± 0.2 µm, Table 5) compared to the base material (w_BM-α-grain_ = 4.5 ± 0.9 µm and w_BM-β-grain_ = 0.6 ± 0.2 µm, Table 5) suggests that this region was thermally affected. Grain growth is a temperature- and time-dependent diffusional process occurring through solute portioning between the α-Ti and β-Ti phases [37]. Therefore, additional energy added through a higher process temperature and additional exposure time is the driving force for the observed grain growth.

Furthermore, no evidence of plastic deformation was detected in Region 1. A macro-geometrical indication of plastic deformation onset in the rivet would be associated with the barreling phenomenon (i.e., increase of rivet diameter [17]). Another indication of a possible micro-scale occurrence of plastic deformation in Region 1 would be the formation of twinning, as shown by Altmeyer et al. [17] for α-commercially pure Ti gr. 3/CF-PEEK friction-riveted joints. However, twins were not detected for the current joints. In general, the formation of some types of twinning in hcp structures (α-Ti phase) is a way to accommodate plastic deformation [38]. For the Ti-6Al-4V alloy, the presence of the cubic crystalline (β-Ti phase) structure may relieve deformations through the larger amount of available slip systems of the bcc structure. Therefore, only thermal phenomena, such as grain coarsening, were observed in Region 1 (Figure 11c).

Region 2 (Figure 11d) is a transition volume in which the rivet experienced less severe thermal processing—probably below the β-transus temperature—cooling and shear rates in comparison to the deformed rivet tip (Regions 3 and 4). The microstructure in this region comprises a mixture of imminent acicular structures (protruding structures indicated by white arrows in Figure 11d) and prior β-grains. In Regions 3 (Figure 11e) and 4 (Figure 11f), the grain morphology was fully transformed into a new microstructure composed of acicular grains. Grain refinement was observed between Region 3 (α-Ti acicular grain size average = 0.5 ± 0.1 µm) and Region 4 (α-Ti acicular grain size average = 0.3 ± 0.06 µm) in comparison to Regions 1 and 2, whereby acicular structures presented narrow α-Ti grains (w/l_Region 3_ = 0.09 ± 0.02 and w/l_Region 4_ = 0.04 ± 0.01, Table 5). These regions are located in the plastically deformed rivet anchoring zone (also characterized by the onset of a barreling effect leading to changes in the original rivet diameter), which experienced the highest strain rates and temperatures during joining. Moreover, the gradient of microstructural changes from Regions 2 to 4 suggests that the cooling rates were inhomogeneous. Similar results were reported by Kitamura et al. [32] in Ti-6Al-4V alloy friction stir welds subjected to similar thermo-mechanical treatment. The measured decrease in aspect ratio from the transition volume (Region 2) to the highly-deformed Regions 3 and 4 are in accordance with their observations; they exhibited a reduction in acicular grain size at high cooling rates as a result of slower growth kinetics. Furthermore, the local temperature in Regions 3 and 4 probably exceeded the non-equilibrium β-transus temperature, as remnant α-Ti primary grains were absent [39].

**Figure 12:**
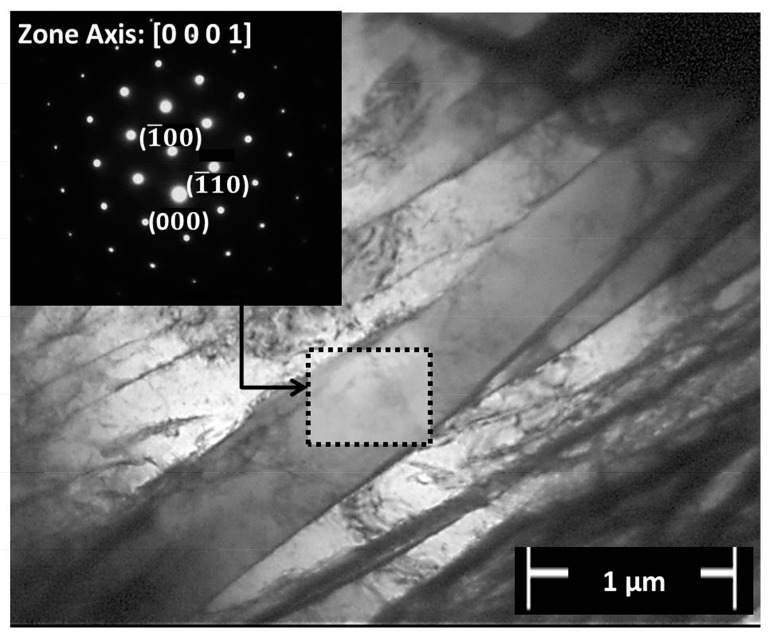
TEM BF image of Widmanstätten microstructure of Ti-6Al-4V with respective SAED pattern oriented in the [0001]α-Ti zone axis from Region 4 of Figure 11f.

Two types of acicular structures were apparently formed in Regions 3 and 4. A pattern of a string-shaped phase with bundles of acicular lamellae growing from this boundary was identified in Region 4 (indicated by a dash-lined rectangle in Figure 11f). According to Baufeld et al. [40], this finding suggests the formation of a typical Widmanstätten structure by a diffusional process. Moreover, an arrow in Figure 11f (in Region 4) indicates acicular phases oriented into geometric patterns; this finding suggests a typical martensitic (α′-Ti) structure formed by a diffusionless transformation [36,41]. TEM analysis was performed to explore these microstructural features. A TEM bright field (BF) image of the lamellae from a Widmanstätten structure identified in Region 4 is shown in Figure 12. The indexed selected area electron diffraction (SAED) pattern displayed hexagonal symmetry attributed to the transformed α-Ti phase oriented in the [0001]α-Ti zone axis.

**Figure 13:**
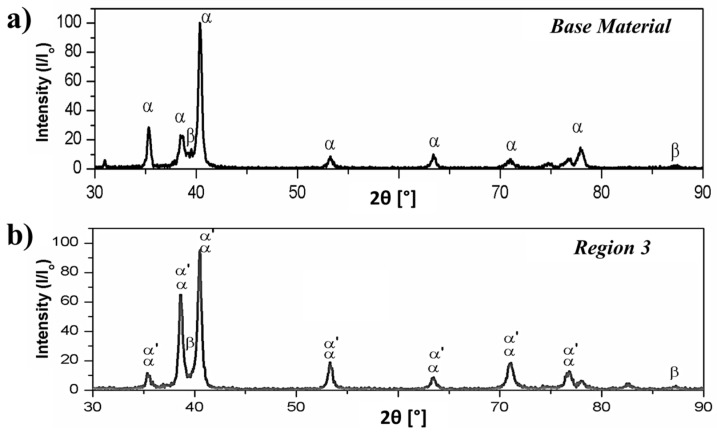
X-ray diffraction patterns of the (**a**) Ti-6Al-4V base material and (**b**) Ti-6Al-4V from the anchoring zone (Region 3, Figure 11b) of the Ti-6Al-4V/GF-P friction-riveted joint.

A comparison between the XRD patterns of the base material and the thermo-mechanically affected sample from Region 3 is in good accordance with the later statement on the martensitic formation. The Bragg’s peaks of the as-received material (Figure 13a) exhibited a simple XRD pattern of hcp α-Ti phase and bcc β-Ti phase. In the XRD pattern of the friction-riveted sample (Figure 13b), the presence of low intensity peaks of the β-Ti phase (at approximately 2θ = 40°) indicated that some of this phase could be converted into the hexagonal martensitic α′-Ti phase. The absence of additional small peaks around the pronounced central peaks of martensite indicates the absence of the orthorhombic martensitic (α″-Ti) phase in Region 3 [42]. This behavior has been also reported by Esmaily et al. [4] for Ti-6Al-4V alloy friction stir and GTAW welds. Therefore, depending on the very local heat input and cooling conditions, either Widmanstätten plate-like or martensitic morphologies were formed, creating a complex microstructure in the anchoring region of the metallic rivet.

**Figure 14:**
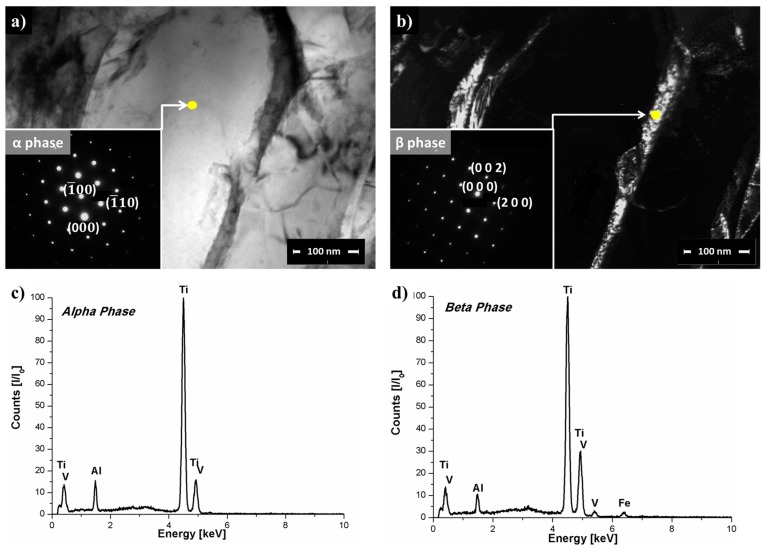
(**a**) TEM BF image of Region 2 from Figure 11b with the respective SAED pattern oriented in the [0001]α-Ti zone axis; (**b**) TEM DF image of Region 2 from Figure 11b detailing the β-Ti phase with SAED oriented in the [001]β-Ti zone axis. EDS spectra showing the chemical composition of the (**c**) α-Ti phase and (**d**) β-Ti phase in Region 2.

TEM-EDS was performed to study the β → α phase transformation regime by estimating the differences in chemical composition between the α- and β-phases of the Ti-6Al-4V base material and in the transition area Region 2 (Figure 11d). Differences in local heating and cooling rates through the rivet length induced inhomogeneous portioning of the alloying element between the phases, resulting in a combination of diffusional and diffusionless processes as discussed in Section 3.3. Figure 14a,b present TEM BF and DF images of Region 2 with respective selected area diffraction pattern (SADP) in the zone axis [0001]α-Ti and in the zone axis [001]β-Ti. Yellow dots in Figure 14a,b indicate the regions where the chemical compositions of α-Ti (Figure 14c) and β-Ti (Figure 14d) phases were analyzed.

No significant changes were observed in the aluminum content of the α-Ti and β-Ti phases in Region 2 of the deformed rivet tip (peak at approximately 1.5 keV in Figure 14b,c) comparing to the base material (peak at approximately 1.5 keV in Figure 3b,c). However, the difference in vanadium content between the α-Ti and β-Ti phases (peak at approximately 5 keV in Figure 14c,d) changed significantly in Region 2 in comparison to the base material. In the base material, the vanadium content in the β-Ti phase was approximately 2.7 times higher (approximate normalized peak intensity = 40) than in the α-Ti phase (approximate peak intensity = 15). In Region 2, the vanadium content in the β-Ti (approximate normalized peak intensity = 30) was only approximately 1.9 times higher than in the α-Ti (approximate normalized peak intensity = 16). This suggests that the vanadium content is more homogeneously distributed between the α-Ti and β-Ti phases in Region 2 in comparison to the base material.

**Figure 15:**
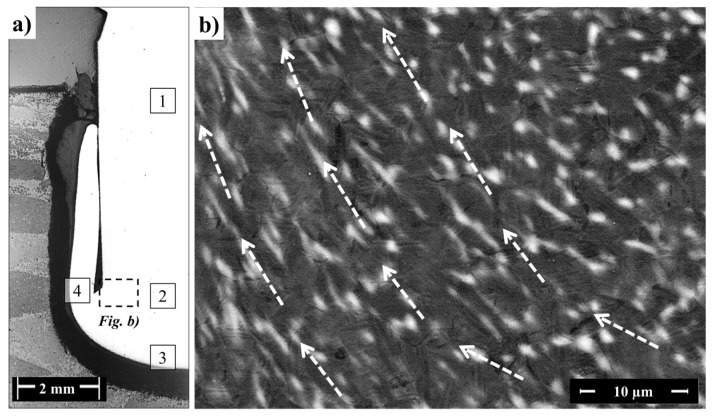
(**a**) Optical image from the cross-section of the HEI joining condition; (**b**) backscattering SEM image of Region 2 near the transition to Regions 3 and 4 showing β-fleck patterns. Arrows in (**b**) indicate the direction of the material flow associated with rivet plastic deformation during the forging phase.

These observations indicated that in this transition area (Region 2), the β → α phase transformation was partially governed by a diffusional process during the cooling of the joint under pressure. Vanadium atoms probably diffused from the β-Ti to the α-Ti phase during the heating phase in FricRiveting because of the high process temperatures (758 ± 56 °C) experienced by the Ti-6Al-4V rivet. During cooling, vanadium diffuses back to the β-Ti phase to stabilize this phase at low temperatures [43]. Nevertheless, because friction riveting involves non-equilibrium thermodynamics, the diffusional process could be considered incomplete, leading to the formation of residual regions saturated with vanadium as presented in Figure 15b by the white speckle pattern. These vanadium-saturated regions are known as β-flecks [27]. β-flecks are usually formed at temperatures above the β-transus temperature, and they appear more commonly for titanium alloys with a greater β-stabilizer amount such as near-β alloys [27].

### 3.4. Local Mechanical Properties

**Figure 16:**
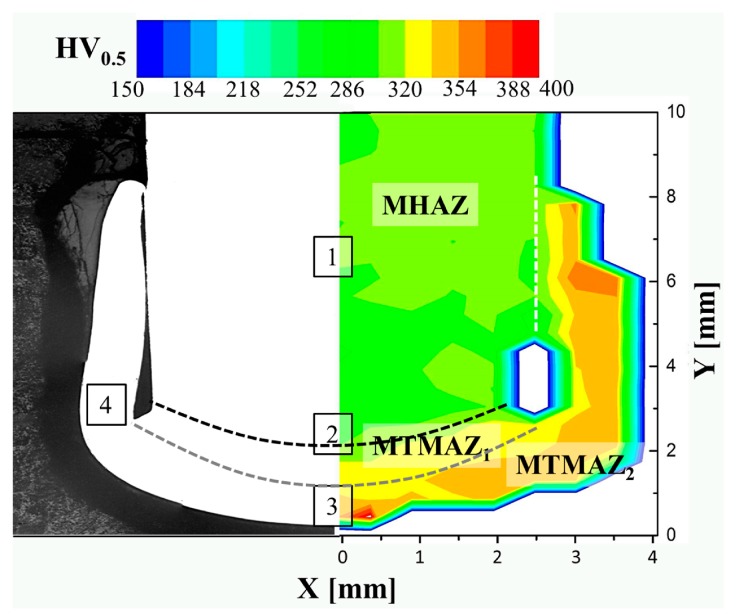
Vickers microhardness map of the Ti-6Al-4V deformed rivet showing the microstructural zones in the rivet from a specimen produced under the HEI joining condition.

The determination of local mechanical properties using a Vickers microhardness map provides complementary support to the previous assumptions on the process-related microstructural changes. Figure 16 exemplifies a microhardness distribution map performed on one half of the metallic rivet cross-section (symmetry assumption) of a HEI joint.

A decrease of 5% to 16% in microhardness (269–286 HV) in comparison to the base material (300–320 HV) was observed in Region 1. This slight decrease in microhardness can be associated with static annealing phenomena such as recovery, discontinuous and continuous subgrain growth, owing to higher process temperatures (but below the β-transus temperature) experienced by the rivet in this volume. This assumption is further supported by the observed increase of 50% in the average grain size of β-Ti grains in Region 1 in comparison to the base material (Table 5). The bcc structure of the β-phase in the equiaxed microstructure of Ti-6Al-4V provides ductility [24]; therefore, the larger β-Ti grains enhance local ductility and decrease microhardness. As no further morphological changes or plastic deformation took place in this region in comparison to the base material (compare with Figure 3a), metallurgical transformations were triggered only by temperature. Therefore, Region 1 can be classified as a heat affected zone (MHAZ), once only static phenomena occur [29].

The highest increase in microhardness was observed at the deformed rivet tip (rivet anchoring zone), where high temperature and plastic deformation induced changes in phase morphology (Regions 2–4, Figure 11d,f). It is possible to subdivide the rivet tip area into two microhardness zones adjacent to the MHAZ. In Zone 1, an increase in microhardness of 5% to 12% (320–337 HV) was observed in comparison to the base material (300–320 HV). This finding can be related to the partial change from equiaxed to acicular grains observed from the imminent needles in Region 2 to Regions 3 (Figure 11d,e) and 4 (Figure 11f), where the microstructure is fully composed of Widmanstätten and α′-martensite structures. Similar behavior was observed by Esmaily et al. [4], who reported a hardness increase in regions affected by high temperatures and plastic deformation in friction stir welded Ti-6Al-4V alloy. Therefore, Zone 1 can be denoted as thermo-mechanically affected zone 1 (MTMAZ1).

Zone 2 is adjacent to MTMAZ1 (encompassing Region 3 and 4) with average hardness increases from 5% to 16% (337–371 HV) compared to MTMAZ1 and 5% to 24% compared to the base material. The formation of a fully acicular microstructure with more refined structures in Region 4 can explain this increase. For instance, the α′-martensitic phase in Ti-6Al-4V was shown to be formed after quenching, with increases in hardness similar to values measured in Zone 2 [44]. Moreover, Esmaily et al. [4], reported that when the process temperature is above the β-transus temperature and the cooling rate is high in FSW, the hardness increases because the acicular average grain size decreases. As this rivet volume was exposed to high plastic deformation rates and cooling rates (Table 4), and the typical α′-martensitic microstructure was observed (Figure 11f), Zone 2 can be denoted as metal thermo-mechanically affected zone 2 (MTMAZ2).

### 3.5. Plastic Deformation in the Rivet Tip

Once the plastic deformation of the rivet tip induces material flow in the joining area, this flow can be further accessed through changes in microtexture by EBSD mapping and the orientation of structures such as β-flecks (described in Section 3.3) formed in the final microstructure.

**Figure 17:**
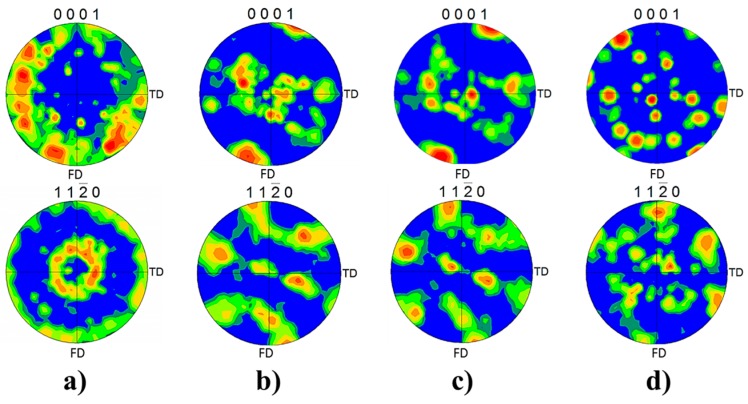
Pole figures from the α-Ti phase for the (**a**) base material; (**b**) Regions 2; (**c**) 3 and (**d**) 4 in the deformed rivet tip (Figure 11b). FD is the forging direction which is analogous to the axial force direction applied during FricRiveting, and TD is the transversal direction.

Although the metallic flow during FricRiveting is complex, the predominant deformation is expected to follow simple-shear texture in a similar manner as described for FSW of titanium alloys [11]. [0001]α-Ti and [11$\bar{2}$0]α-Ti pole figures were chosen to evaluate the simple-shear texture imposed by FricRiveting for each region of interest in the metallic rivet (Regions 2 to 4 in Figure 11b). Figure 17 shows the respective EBSD pole figures. Considering that the fractions of the retained β-Ti phase were too low in the investigated regions—as shown in the X-ray spectrum of Figure 13b—and the β → α phase transformation follows the Burges orientation relationship [101]β-Ti∥[0001]α-Ti and [11$\bar{1}$]β-Ti//[11$\bar{2}$0]α-Ti, current pole figures from the α-Ti phase can be used for qualitative texture analysis only [10].

Texture measurement results reveal a decrease in microtexture from the base material to Region 4. The basal planes [0001]α-Ti in Regions 2 (Figure 17b) and 3 (Figure 17c) were re-oriented at approximately 45° to the forging direction FD (analogous to the axial force direction applied during the forging phase of FricRiveting) probably because of the higher shear stresses. In Region 4 (Figure 17d), the microstructure apparently lacks microtexture, which can be associated with the fully acicular microstructure (Figure 11f). The lack of deformation texture can be attributed to the number of possible orientations by which the β-Ti grains can potentially transform into the α-Ti phase. A single bcc grain can transform, in theory, into 12 possible orientations or variants during β → α phase transformation because of the number of available slip systems in the α-Ti phase [45]. Nevertheless, one should bear in mind that the current assumption only partially explains the randomized final texture, as the 12 possible slip systems orientations or variants have the same statistical probability of forming.

In the current investigation, continuous dynamic recrystallization (CDRX) was not directly identified in the microstructure of the plastically deformed rivet volume (Regions 3 and 4, Figure 11e,f). Therefore, this assumption cannot be taken into account to explain the random grain orientation in Figure 17d. This finding contradicts typical observations for Ti-6Al-4V alloy in friction-based joining processes [37]. In CDRX of Ti-6Al-4V alloy, colonies of lamellae can usually be easily recrystallized because they are in a non-ideal orientation, storing energy faster (because of the frictional process) to spheroidize [46]. Thus, current results suggest a strong effect of the β → α phase transformation and morphology changes on the microtexture of the rivet tip, which was a result of the induced material flow during the plastic deformation regime in the metallic rivet. Finally, the presence of preferential orientation of the β-flecks in Region 2 (represented by the white speckle pattern in Figure 15b) helps support the occurrence of simple shear regimes inducing the “leg geometry” in the deformed rivet tip. This preferential orientation reveals the material response to the imposed forging forces and induced plastic deformation, as β-flecks seems to have been deformed and realigned in the direction of the rivet deformation flow during the forging phase of the joining process (see the auxiliary dash-lined arrows in Figure 15b).

## 4. Conclusions

Friction-riveted joints of glass-fiber reinforced polyester and Ti-6Al-4V alloy, exhibiting quasi-static lap shear strength comparable to state-of-the-art bolted connections were investigated in the current work. Two levels of energy input (low energy input specimens = 1750 ± 170 J and high energy input specimens = 2000 ± 250 J) were selected, resulting in process temperatures varying from 460 ± 130 °C to 758 ± 56 °C. Microstructural changes suggest that these process temperatures overcame the non-equilibrium β-transus temperature of the Ti-6Al-4V alloy in the anchoring region where the metal was highly affected by the heat and the plastic deformation. Moreover, high cooling rates were measured for both joining conditions (178 ± 15 °C/s and 59 ± 15 °C/s) which can be explained by the high process temperatures and by the thermal isolation effect related to the low conductivity of the polyester composite.

The combination of thermo-mechanical treatment and complex cooling regimes resulted in a bi-modal microstructural gradient in the rivet. Outside the region where rivet tip widening occurred, equiaxed morphology similar to the base material was observed, and no evidence of plastic deformation was identified. Moreover, the occurrence of thermal phenomena such as β-Ti grain growth may explain the decrease of up to 16% in microhardness in this region in comparison to the base material. These features allowed denoting this region as a heat affected zone. In the deformed rivet tip, a fine acicular microstructure was formed as a result of higher local temperature (superior to the non-equilibrium β-transus temperature), faster cooling and shear rates. The acicular microstructure revealed the presence of α′ martensite (diffusionless phase transformation) along with Widmanstätten structures (diffusional phase transformation). This observation was confirmed by scanning and transmission electron microscopy, supported by analysis of CCT diagrams, and X-ray diffraction patterns. Incomplete diffusional processes were apparent in this plastically deformed region from the presence of regions enriched with vanadium element (β-flecks). No continuous dynamic recrystallization was identified. Current results suggest that a process-related thermo-mechanical effect induces a β → α phase transformation and morphology changes hindering the onset of dynamic recrystallization. Furthermore, the formation of this complex microstructure strongly affected the local mechanical properties, leading to an increase of up to 24% in the microhardness of the deformed rivet volume in comparison to the base material. Facing the gradual morphology transformation in the rivet tip, from equiaxed (in the heat affected zone) to thin acicular morphology, two thermo-mechanically affected zones were defined according to the intensity of the phase transformation and the corresponding increase in microhardness.

The deformational material flow in the rivet tip was studied by analyzing the microtexture of the α-phase and the final orientation of the formed β-flecks domains. EBSD pole figure results indicated the orientation of the grains to be 45° to the forging direction and a loss of microtexture in the plastic deformed rivet volume, an indication of a simple shear regime. This change in microtexture is a result of the thermo-mechanical influence on the rivet tip and the β → α phase transformation (grains assuming a complex acicular morphology, orientation and average size). Furthermore, the orientation of β-flecks in the highly-deformed volumes of the rivet tip indicates the material response to the imposed forging forces and induced plastic deformation, leading to the widening of the rivet tip. Therefore, this study provided, for the first time, a detailed investigation on the relationships between microstructural transformations, changes in local mechanical properties and deformational material flow in friction-riveted joints of Ti-6Al-4V and glass-fiber-reinforced thermosets. The acquired understanding is a key tool to support future computational modelling of rivet plastic deformation. The results of this study may provide a base for further understanding of the joint formation and thus improvement of the joint mechanical performance.

## Figures and Tables

**Table 1:** Experimental chemical composition of Ti-6Al-4V alloy rivets.

**Weight (wt. %)**	**N**	**H**	**O**	**Fe**	**Al**	**V**	**Ti**
	0.002	0.003	0.107	0.217	6.2	4.5	Bal.

**Table 2:** Selected thermal and mechanical properties of AA 2198-T8 [23], Ti-6Al-4V alloy [24] and of glass-fiber-reinforced polyester (GF-P) [22].

Base Material	Tensile/Shear Strength (MPa)	Hardness (HV)	Β-transus Temp., T_β__-transus_ (°C)	Melting Temp., T_m_ (°C)	Glass Transition Temp., T_g_ (°C)	Degradation Temp., T_d_ (°C)	Thermal Conductivity (W/m·K)	Coefficient of Thermal Expansion within 20–650 °C (µm/m·°C)
AA 2198-T8	436–510/296	180	-	567	-	-	-	-
Ti-6Al-4V	940–1180/550	320	995	1655	-	-	17.5	9.7
GF-P	240/100	50–83	-	-	60–80	390	0.25–0.35	9.0

**Table 3:** Joining conditions and calculated energy input for the specimens investigated in this work.

Joining Conditions	Friction Time (s)	Forging Time (s)	Rotational Speed (rpm)	Friction Pressure (MPa)	Forging Pressure (MPa)	Energy Input (J)
**Low Energy Input (LEI)**	1.0	1.2	9000	0.6	1.0	1750 ± 170
**High Energy Input (HEI)**	1.2	1.2	9000	0.6	1.0	2000 ± 250

**Table 4:** FricRiveting process temperatures and cooling rates for Ti-6Al-4V alloy/GF-P specimens produced under LEI and HEI conditions.

	LEI	HEI
**Average Peak Temperature (°C)**	460 ± 130	758 ± 56
**Cooling Rate (°C/s)**	178 ± 15	59 ± 15

**Table 5:** Grain sizes and aspect ratios of different microstructures developed through the rivet during FricRiveting.

	Base Material	Region 1	Region 2	Region 3	Region 4
	Equiaxed Microstructure		Transition	Acicular Microstructure	
**α-Grain Width, w (µm)**	4.5 ± 0.9	5.4 ± 1.8	4.9 ± 1.0	0.5 ± 0.1	0.3 ± 0.06
**α-Aspect Ratio, w/l**	0.9 ± 0.02	0.9 ± 0.1	0.8 ± 0.1	0.09 ± 0.02	0.04 ± 0.01
